# Potentially Toxic Element Contaminations and Lead Isotopic Fingerprinting in Soils and Sediments from a Historical Gold Mining Site

**DOI:** 10.3390/ijerph182010925

**Published:** 2021-10-18

**Authors:** Lei Tang, Yiyue Zhang, Shuai Ma, Changchun Yan, Huanhuan Geng, Guoqing Yu, Hongbing Ji, Fei Wang

**Affiliations:** 1 School of Energy & Environmental Engineering, University of Science and Technology Beijing, 30 Xueyuan Road, Beijing 100083, China; tangtom1220@163.com (L.T.); yiyue.zhang@hotmail.com (Y.Z.); shuai.ma@yale.edu (S.M.); y19801286607@163.com (C.Y.); genghuanhuan0325@outlook.com (H.G.); 2 Beijing Key Laboratory of Resource-Oriented Treatment of Industrial Pollutants, 30 Xueyuan Road, Beijing 100083, China; 3 Beijing Geo-Exploration and Water Environment Engineering Institute Co., Ltd., 9 Linglong Road, Beijing 100142, China; 13366833331@163.com

**Keywords:** miyun reservoir, pollution assessment, binary mixing model, source appointment

## Abstract

Lead (Pb) isotopes have been widely used to identify and quantify Pb contamination in the environment. Here, the Pb isotopes, as well as the current contamination levels of Cu, Pb, Zn, Cr, Ni, Cd, As, and Hg, were investigated in soil and sediment from the historical gold mining area upstream of Miyun Reservoir, Beijing, China. The sediment had higher ^206^Pb/^207^Pb ratios (1.137 ± 0.0111) than unpolluted soil did (1.167 ± 0.0029), while the soil samples inside the mining area were much more variable (1.121 ± 0.0175). The mean concentrations (soil/sediment in mg·kg^−1^) of Pb (2470/42.5), Zn (181/113), Cu (199/36.7), Cr (117/68.8), Ni (40.4/28.9), Cd (0.791/0.336), As (8.52/5.10), and Hg (0.168/0.000343) characterized the soil/sediment of the studied area with mean *I_geo_* values of the potentially toxic element (PTE) ranging from −4.71 to 9.59 for soil and from −3.39 to 2.43 for sediment. Meanwhile, principal component analysis (PCA) and hierarchical cluster analysis (HCA) coupled with Pearson’s correlation coefficient among PTEs indicated that the major source of the Cu, Zn, Pb, and Cd contamination was likely the mining activities. Evidence from Pb isotopic fingerprinting and a binary mixing model further confirmed that Pb contamination in soil and sediment came from mixed sources that are dominated by mining activity. These results highlight the persistence of PTE contamination in the historical mining site and the usefulness of Pb isotopes combined with multivariate statistical analysis to quantify contamination from mining activities.

## 1. Introduction

In the past few decades, potentially toxic element (PTE) contaminations from mining activities have become a serious global environmental problem [1,2,3,4]. Mining activity constitutes prominent sources of toxic, corrosive, radioactive, or nonradioactive metal contaminants from ore, smelting, mineral dressing, and the erosion of mine tailings [5]. Both historical and ongoing mining activities have a nonnegligible impact on the surrounding environment, resulting in significant increases in PTE loads in both aquatic and terrestrial ecosystems [6]. The release of large amounts of mine wastes from mining and transportation, acid mine drainage (AMD) from ore mineral dressing, and fly ash, as well as particulate matter, from metal smelting and coal combustion all potentially lead to significant increases in PTE loads in the surrounding environment. Previous works have found strong evidence of PTE contamination in the soils, water, sediments, atmosphere, and biota in proximity to mining activities [2,7,8,9,10]. Identifying anthropogenic sources of PTEs and the apportionment of the contributions of anthropogenic and natural sources has caused significant concern because it is of crucial importance to preventing and controlling PTE contamination [11,12]. Although the contamination of the surrounding environment by PTEs from mining activities has been extensively studied and highlighted, source interpretation of mining-impacted areas remains challenging, especially in historical small-scale polymetallic mining sites [2].

Multivariate statistical analysis is a traditional and useful tool to identify potential factors that may indicate or hint to sources of PTE concentration and to explore similarities and hidden patterns among the sample [13,14]. Principal component analysis (PCA) and cluster analysis (CA) are common widely used techniques and are often combined to identify the potential sources of PTEs in soils and sediments [15]. The multivariable statistics analysis of PTE concentration provides vital information on the interrelationships of elements. However, its source identification and apportionment usually rely strongly on statistical approaches, which have required large databases and sophisticated statistics [16]. Pb isotope fingerprinting has shown great advantages in the identification and quantification of various sources in environmental studies [17,18]. The four natural Pb stable isotopes (^204^Pb, ^206^Pb, ^207^Pb, and ^208^Pb) in natural or anthropogenic origin sources (e.g., ore deposits, coal, and leaded gasoline) typically have their unique signatures and result in the distinguishable Pb isotope ratio [13,16,19]. No significant Pb isotopic fractionation occurs during natural and anthropogenic processes, implying that the final Pb isotopic composition in the environment reflects only the original source of Pb or a mixture of multiple sources, thus allowing us to evaluate the contributions from the different Pb sources [16,19,20,21]. Studies are increasingly using Pb isotope fingerprinting to trace the anthropogenic Pb sources in sediments, soils, coal fly ash, aerosols, and other environment archives [11,12,22,23,24,25]. Recently, Pb isotopes have been employed to trace sources of gold deposits [26].

Miyun Reservoir (40°31′ N, 116°51′ E) is the largest reservoir and the primary surface drinking water source for Beijing with a population exceeding 20 million [27,28]. It has a storage capacity of 438 GL. Bai and Chao River are the leading natural replenishments of Miyun Reservoir that contribute mean flows of 111 GL yr^−1^ and 203 GL yr^−1^, respectively [29]. Historically, long-term small-scale metal (gold, iron, copper, etc.) mining and smelting activities have had a nonnegligible impact on the environment surrounding the Miyun Reservoir [27]. Since 2005, numerous small-scale metal mine sites have been closed in this area. However, mine waste from mining operations is still deposited in abandoned tailings ponds, continuing to cause PTE contamination to the neighboring environment. Several studies have reported that the PTE contamination of soils, river sediments, and river water upstream of Miyun Reservoir is mainly caused by mining activities [27,30,31,32,33,34]. Some other studies have concluded that coal combustion and vehicle exhaust were identified as the primary source of PTEs in surface soils. It is difficult to infer the contribution of various sources from the elevated concentration of PTEs, especially in the mining area [35]. In addition, the levels and sources of PTEs in sediments have rarely been reported in this area compared to soils (Zhu et al., 2013), even though sediment is the appropriate indicator of PTEs in aquatic systems [36].

To investigate the impact of mining activities on the accumulation of PTEs in the surrounding environment, Pb isotopes, as well as the current contamination status of eight typical PTEs (Cu, Pb, Zn, Cr, Ni, Cd, As, and Hg) in soils and sediments, were determined surrounding the mining-impacted area upstream of Miyun Reservoir, Beijing, China. We aimed (i) to assess the pollution of PTEs; (ii) to identify and appoint the potential pollution sources using Pb isotope fingerprinting and multivariate statistical analysis; (iii) to quantify the Pb contribution from the potential sources using stable isotope mixing models.

## 2. Materials and Methods

### 2.1. Sample Collection and Preparation

**Figure 1:**
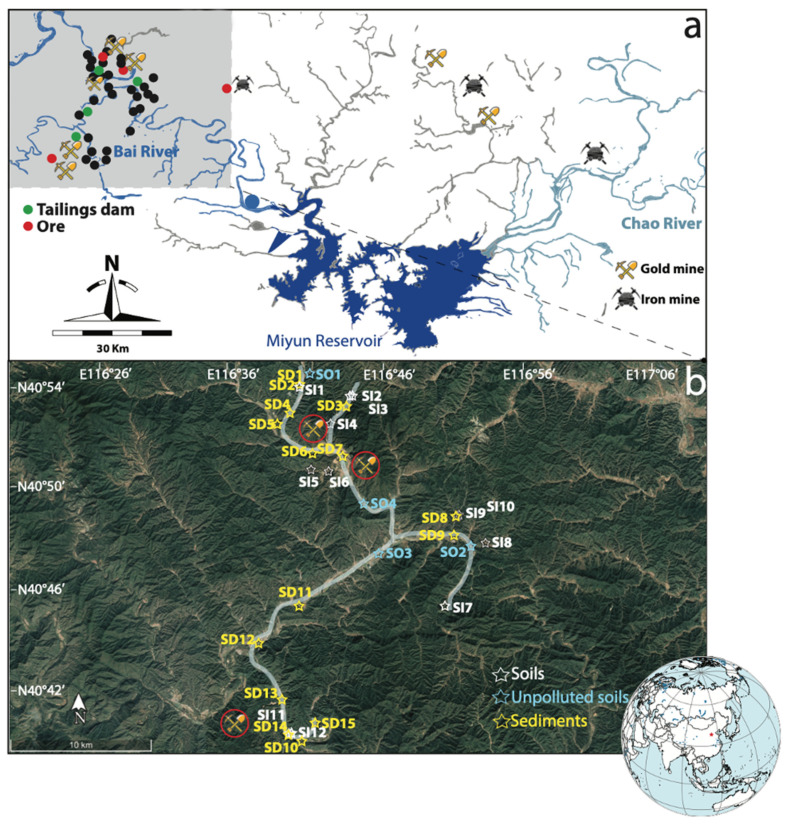
Map of the upstream of Miyun Reservoir (**a**); soils and sediments sampling sites (**b**).

As shown in Figure 1, a total of 35 sampling sites (three points at each site) were taken from the small-scale gold ores scatter areas upstream of the Miyun Reservoir, including 16 surface soil sites, 15 surface sediments sites (SD1–SD15), and four tailings dam sites. Of the 16 soil sampling sites, 12 sites (SI1–SI12) were taken from an area heavily impacted by mining activities in proximity to local and regional potential contaminant sources (e.g., mines and tailings dams), and four unpolluted sites (SO1–SO4) were taken from woodlands, villages, and agricultural lands that were far from the mining activities and had not been strongly impacted. In 2005–2013, mining activities in the sampling area were completely abandoned due to local government policies.

### 2.2. Chemical Treatments and Analysis

Samples were first freeze-dried and sieved through the 200 mesh (<0.074 mm) stainless-steel sieve. Subsamples (0.1 g) were added with 4 mL of concentrated HNO_3_ and 0.5 mL of H_2_O_2_ (30%) in a Teflon beaker before heating at 90 °C overnight until dryness. The samples were further digested with 2 mL of concentrated HNO_3_, 2 mL of HF, and 1 mL of HClO_4_ in a sealed beaker at 120 °C for 48 h. Upon evaporation until dryness, re-dissolved in 5% HNO_3_, the digester was measured for total element concentration. Standard reference materials were processed with the same digestion procedure. Pb isotopic analyses (^204^Pb,^206^Pb, ^207^Pb, and ^208^Pb) were determined in a selection of soils and sediment samples using a Nu Instruments HR^®^ double focusing MC-ICP-MS. Samples were calibrated against the National Institute of Standards and Technology (NIST) SRM 981 standard after each sample measurement. The measured isotopic ratios of the standard NIST SRM 981 were ^204^Pb/^206^Pb = 0.059 ± 0.001 (2SD), ^206^Pb/^207^Pb = 1.093 ± 0.002 (2SD), and ^208^Pb/^206^Pb = 2.166 ± 0.003 (2SD), which had good agreement with their respective certifications 0.059, 1.093, and 2.168 [17].

### 2.3. Quality Assurance and Quality Control (QA/QC)

The lab glassware and Teflon beakers were previously soaked in 50% HNO_3_ (*w*/*w*) at 120 °C for at least 48 h, followed by rinsing with 18.2 MΩ/cm of Milli-Q water before usage. All analytical solutions were executed in triplicate, and the result was expressed as the mean value. The quality of the processing and analytical procedures was tested by measurements on the Chinese national geo-standard (GBW-07333 and GBW-07314) provided by the National Research Center for certified Reference Materials of China. The standard solutions (NIST) SRM 981 were measured after every ten samples in the analysis of PTE concentrations and after every signal sample in the analysis of Pb isotopic ratio. Instrument performance and analytical procedure reproducibility were determined by analyzing the United States Geological Survey (USGS) reference materials BCR-2 (Basalt, Columbia River) and AGV-2 (Andesite, Guano Valley). The BCR-2 standard resulted in ^206^Pb/^207^Pb = 1.209 ± 0.006 (2SD) and ^208^Pb/^206^Pb = 2.065 ± 0.003 (2SD), in agreement with the values reported [37]. The AGV-2 resulted in ^206^Pb/^207^Pb of 1.208 ± 0.001 (2SD) and ^208^Pb/^207^Pb of 2.468 ± 0.008 (2SD), also in agreement with previously published values [38]. The standard error of ^207^Pb/^206^Pb measurements was less than 0.3% RSD. The results of PTE concentrations were consistent with all reference values, and the differences were within ±7%. The relative error was lower than 10%, and the relative standard deviation (RSD) was lower than 5% for all tests.

### 2.4. Pollution Risk Assessment

The contamination factor (*CF*), degree of contamination and pollution load index (*PLI*), geo-accumulation index (*I*_geo_), and potential ecological risk assessment (*RI*) were determined to assess the potential extent of PTE contamination in different sampling sites.

#### 2.4.1. Contamination Factor (*CF*)

*CF* was used to assess environmental contamination caused by an excess of a single metal in a sample by calculating the ratio of the measured metal concentration to the natural background value of the metal [39], calculated according to Equation (1).

(1) $${CF}_{i}=C_{f}^{i}=C_{m}^{i}/C_{b}^{i}$$

where $C_{m}$ represents the measured concentration of metal *i*, and $C_{b}$ represents the reference value for metal *i*. The reference value used here was the background value (BV) for PTEs in natural soils in Beijing [40]. Based on the calculated *CF* values, the degree of contamination was divided into four levels: low (*CF* < 1); moderate (1 ≤ *CF* < 3); considerable (3 ≤ *CF* < 6); and very high (*CF* ≥ 6).

#### 2.4.2. Pollution Level Index (*PLI*)

The pollution load index (*PLI*) was used to assess the overall combined toxicity to the environment at each sampling site by standardizing the contribution of all the evaluated PTEs [41]. *PLI* was calculated as the nth root of the product of contamination factors ($CF_{i}$), calculated according to Equation (2).

(2) $$PLI=\sqrt[{\;n}]{CF_{1}\times CF_{2}\times \cdots CF_{i}\times \cdots CF_{n}}$$

where n is the sum number of evaluated PTEs. *PLI* classifies six classes of metal contamination from low to high as follows [41]: unpolluted (*PLI* ≤ 1); unpolluted to moderate (1 < *PLI* ≤ 2); moderately polluted (2 < *PLI* ≤ 3); moderately to highly polluted (3 < *PLI* ≤ 4); highly polluted (4 < *PLI* ≤ 5); and very highly polluted (*PLI ≥* 5).

#### 2.4.3. Geo-Accumulation Index (*I*_geo_)

The commonly used *I*_geo_ is a geochemical criterion proposed by Muller [42] to quantify metal contamination from natural activities (geological and geographical processes) and anthropogenic activities in soils or sediments, calculated according to Equation (3).

(3) $$I_{\mathrm{geo}}=\log_{2}\frac{C_{m}}{1.5C_{b}}$$

The constant 1.5 is introduced as the background matrix correction factor for lithospheric effects. Igeo classifies seven classes of metal contamination from low to high as follows [43]: unpolluted (*I*_geo_ < 0); unpolluted to moderately polluted (0 ≤ *I*_geo_ < 1); moderately polluted (1 ≤ *I*_geo_ < 2); moderately to heavily polluted (2 ≤ *I*_geo_ < 3); strongly polluted (3 ≤ *I*_geo_ < 4); strongly to extremely polluted (4 ≤ *I*_geo_ < 5); and extremely polluted (*I*_geo_ ≥ 5).

#### 2.4.4. Potential Ecological Risk Assessment

The potential ecological risk index of an individual element ($E_{r}^{i}$) and the comprehensive potential ecological risks (RI) and of PTEs in soils and sediments were evaluated following Equations (1), (4) and (5), as established by Hakanson [41].

(4) $$E_{r}^{i}=C_{f}^{i}\times T_{r}^{i}$$

(5) $$\mathrm{RI}=\sum^{}E_{r}^{i}=\sum^{}(C_{f}^{i}\times T_{r}^{i})$$

$T_{r}^{i}$ is the toxicity response factor of each metal, where Hg = 40, Cd = 30, As = 10, Cu = Pb = Ni = 5, Cr = 2, and Zn = 1 [44,45]. $C_{f}^{i}$ is calculated as Equation (1). The ecological risks of individual metal ($E_{r}^{i})\;$were divided into five levels: low risk, ($E_{r}^{i}$ < 40); moderate risk (40 ≤ $E_{r}^{i}$ < 80); considerable risk (80 ≤ $E_{r}^{i}$ < 160); high risk (160 ≤ $E_{r}^{i}$ < 320); and very high risk ($E_{r}^{i}\;$ > 320). Based on RI values, the comprehensive ecological risks of PTEs were divided into four levels: low risk (RI < 150); moderate risk (150 ≤ RI < 300); considerable risk (300 ≤ RI < 600), and high risk (RI > 600).

### 2.5. Data Analysis

XSLTAT software and R (version 3.6.3) were used for the statistical analysis, including Pearson’s correlation analysis, HCA, and PCA. The statistical method was performed with a 95% confidence interval (significance *p* < 0.05). Due to a wide range of Pb and other metal concentrations in soils and sediments, the original data were standardized before carrying out HCA and PCA [15].

A binary mixing model was used to quantify the relative contributions of mining activity-associated Pb to the soils and sediments. The model was calculated from the values of ^206^Pb/^207^Pb and ^208^Pb/^206^Pb and the mean contribution was derived [46], calculated according to Equations (6)–(8).

(6) X1=(P206bP207b)Sample−(P206bP207b)Source A(P206bP207b)Source A−(P206bP207b)Source B

(7) X2=(P208bP206b)Sample−(P208bP206b)Source A(P208bP206b)Source A−(P208bP206b)Source B

(8) $$\bar{X}=\left(X_{1}+X_{2}\right)/2$$

where $X_{1}$ and $X_{2}$ are the percentages fraction of source A calculated from ^206^Pb/^207^Pb and ^208^Pb/^206^Pb, respectively.$\;\bar{X}$ is the average of $X_{1}$ and $X_{2}$.

## 3. Results and Discussion

### 3.1. Current PTEs Contamination in Soils and Sediments

The average concentrations of the studied PTEs in soils and sediments, in order of abundance, were as follows: Pb > Cu > Zn > Cr > Ni > As > Cd > Hg and Zn > Cr > Pb > Cu > Ni > As > Cd > Hg, respectively (Table 1). In general, the PTE concentration of unpolluted soils (outside mining area, SO1–SO4) was close to the background value of Beijing (Table 1), consistent with the previous studies for forest and grassland soils of Miyun Reservoir [30] and rural soils of Beijing [15]. The concentrations of all investigated PTEs were significantly higher in the mining-impacted soils (SI1–SI12) than in the unpolluted soils (SO1–SO4). For example, the mean concentration of Cd in the unpolluted soils (SO1–SO4) (0.13 mg·kg^−1^) was similar to the Cd geochemical baseline concentration (0.12 mg·kg^−1^) in Miyun Reservoir [47], whereas up to 0.79 mg·kg^−1^ (mean value) of Cd was detected in the mining-impacted soils (SI1–SI12). The mean concentrations of Cu, Pb, Zn, Cr, Ni, Cd, As, and Hg in the sediments were 35, 39, 94, 67, 28, 0.3, 4.4, and 0.03 kg^−1^, respectively, which are comparable to the levels in other river sediments in China [48,49]. However, the concentrations (in mg·kg^−1^) of Zn (228), Pb (192), and Cu (92.1) were much higher than those of the upper continental crust [50] and Beijing background values [16]. Therefore, it can be predicted that metal-rich ores may be responsible for the significant increase in the concentration of some specific PTEs (Pb, Cu, and Cd) in the river sediments [48,51].

**Figure 2:**
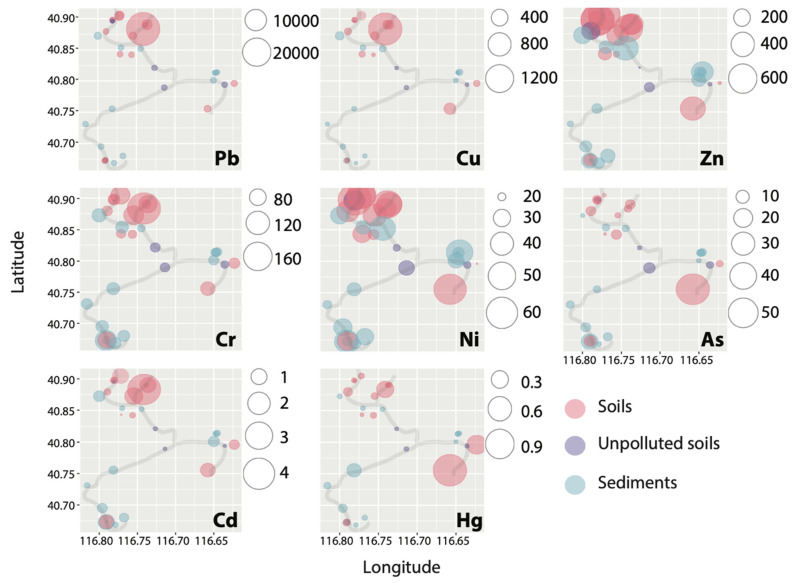
Spatial distribution of PTE contents (mg/kg) in soils and sediments.

Spatially, the PTEs in the soils (32% < CV < 220%) were considerably more variable than in the sediments (29% < CV < 129%), although both were significantly higher than in unpolluted soils (10% < CV < 45%), as shown in Figure 2 by different colors. The variations may be caused by the complex geological and geographical features among the different sites and the surrounding anthropogenic activities [51]. Extremely high concentrations (in mg·kg^−1^) of Pb (27,368), Cu (1582), Zn (792), and Cd (4.1) were found at Site SI6, which is geographically adjacent to the ore deposits (Figure 1 and Figure 2). Cr (195) and Ni (62.3) exhibited the highest concentration at Site SI2, and the highest concentration of As (59.1) and Hg (1.14) was found at SI11. It can be seen from Table 1 that the maximum concentrations of each PTE were all detected in the soils (SI2, SI6, and SI11), and the mean concentrations of PTEs in the soils were also greater than in the sediments. Therefore, the overload of PTEs in soils and sediments is likely to have been disturbed by varying degrees of mining activities. Meanwhile, the soils in the study area were more significantly disturbed by mining activities. On the one hand, this phenomenon may originate from the fact that soils inside the mining area are closer to the source(s) point of contamination from various mining activities (Figure 1). The distance between sampling sites and point sources (e.g., mining, smelters, and tailings dams) may significantly affect metal accumulation through different diffusion intensities of anthropogenic activities [52,53]. On the other hand, the mobility and availability of PTEs in soils and sediments are influenced by several factors, including topography, oxic–anoxic interface, adsorption/desorption processes, pH, salinity, and organic matter [54]. The results of the current metal contamination in soils and sediments further confirm previous findings that there are varying degrees of metal contamination upstream of the Miyun Reservoir [27,33].

### 3.2. Pollution Assessment of PTEs in Soils and Sediments

**Figure 3:**
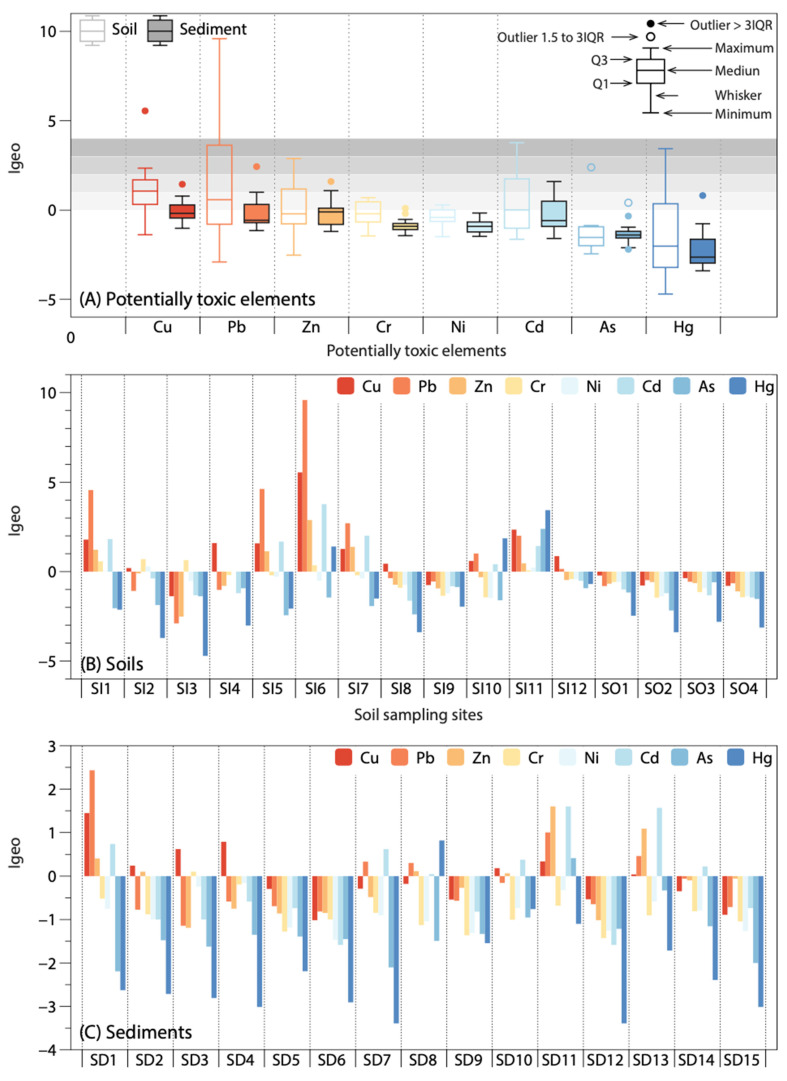
*I_geo_* of PTEs in soils and sediments.

The pollution assessment methods, including *CF*, *PLI*, *I*_geo_, and *RI*, generally reflected higher contamination levels in the soils than in the sediments, as shown in Figure 3 and Table 2. For example, average *CF* values of Cu, Pb, Zn, Cr, Ni, Cd, As, and Hg in sediments were 1.63, 1.79, 1.58, 0.86, 0.85,1.68, 0.68, and 0.49, respectively, which belong to the unpolluted (*CF* < 1) and moderate-to-low (1 ≤ *CF* < 3) polluted levels (Table 2). However, the average *CF* values of PTEs in soils were much higher with very high polluted levels (*CF* ≥ 6) of Cu (8.84) and Pb (8.80); high polluted levels of Cd (3.96); and moderate-to-low polluted levels of Zn (2.53), Cr (1.46), Ni (1.19), As (1.14), and Hg (2.40). The *PLI* values of the sediment sites ranged from 0.57 to 1.92 with an average of 0.97, indicating almost no heavy pollution (*PLI* > 1), while seven of the 12 soil sampling sites had *PLI* values within 1.2–9.8 (Table 2). Similarly, the mean *I*_geo_ value for each PTE in the sediments was below zero, while the mean *I*_geo_ values in soils for PTEs ranged from −1.37 to 1.56 (Figure 2). The difference in *I*_geo_ between soils impacted by mining activities (SI1-SI12) and unpolluted soils (SO1–SO4) can be clearly seen in Figure 2, with *I*_geo_ values as high as 9 for the former and below 0 for the latter. Notably, for Pb, the Igeo values for SI1, SI5, and SI6 were classified as strongly contaminated (4 ≤ *I*_geo_ < 5), and for SI6, it was classified as extremely contaminated (*I*_geo_ ≥ 5). Notably, the highest *PLI* value was found at SI6 (9.8), followed by SI11 (4.4), SI1 (2.5), and SI5 (2.1), all located within 2 km of either of the tailings ponds, mines, or the smelters (Figure 1); thus, proximity to mining activities might account for the higher *PLI* at these soil sites. None of the PTEs in sediments showed a heavy or extreme pollution index with *I*_geo_ < 3. Sediment sampling sites did not have high or very high contamination indices, and most sampling sites were uncontaminated to moderately contaminated with *PLI* < 2, *CD* < 20, and *RI* < 300 (Table 2). One-third of the sediment sampling sites had uncontaminated levels (*I*_geo_ < 0) for all PTEs, indicating that these sites (SD5, SD6, SD9, SD12, and SD15) have not been impacted by mining activities.

### 3.3. Source Identification

**Figure 4:**
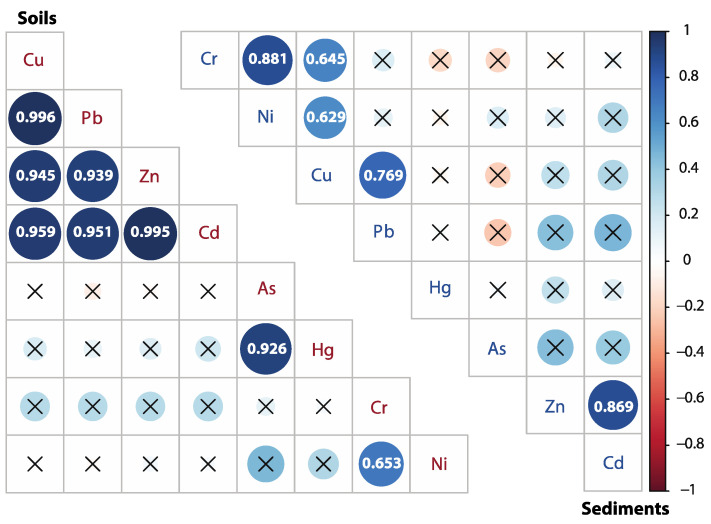
Pearson’s correlation analysis of PTE concentration in soils (**Left**) and sediments (**Right**) inside the mining area.

**Figure 5:**
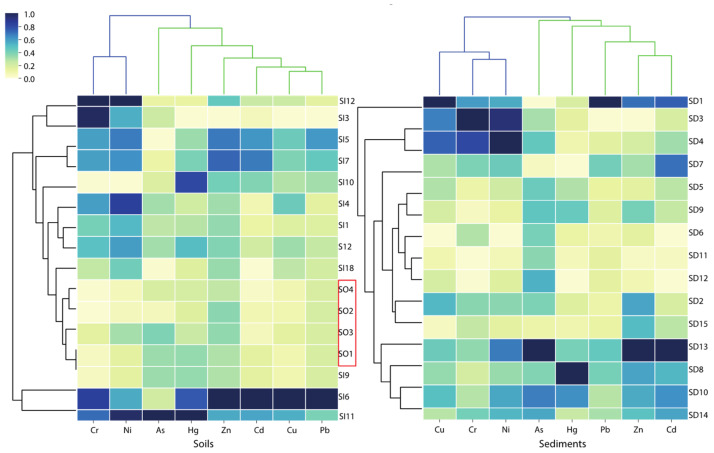
The HCA results are shown as 2D (Euclidean distance; agglomeration method: Ward’s method).

Pearson’s correlation coefficient (Figure 4) showed a strongly positive correlation between Pb with Cu (0.996), Zn (0.939), and Cd (0.995) in the soils (*p* < 0.01). Meanwhile, As and Hg (0.926) had a significantly positive correlation in the soil, as well as Cr and Ni (0.653). The results of PCA (Table 3) displayed that factor one (F1) captured Cu, Pb, Zn, Cd, and Hg (52.7%), and factor two (F2) captured Cr and Ni (23.4%) in the soils, accounting for 76.2% of the total variance. This evidence indicates that they have possible parallel geochemical behaviors, which means they are likely from the same source(s) [33,55,56]. HCA was used to group sample sites and PTE concentrations together (shown as 2D heatmap), which provides more information in terms of the point source distribution and potential sources in soils and sediments. As shown in Figure 5, the unpolluted soils (SO1–SO4) clustered together and exhibited relatively low levels of PTEs. With the exception of sample site SI9, all other soils inside the mining area exhibited contamination with different PTEs. For example, SI5, SI6, and SI7 exhibited relatively high levels of Pb, Zn, Cu, and Cd. In addition, SI11 showed high levels of Hg, As, Ni, and Cr. These pieces of evidence combining the higher loading of PTEs than the background value of Beijing pointed out that Cu, Pb, Zn, and Cd in the soils inside the mining area likely originated from mining activities. The results confirm previous research that mining activities are the major source of Cu, Zn, and Pb from Pb-Zn ores, atmosphere deposition, acidic mine drainage wastewater from smelters, erosion, and leaching of tailings [57,58]. In addition, Ni and Cr in soils were closely associated with natural sources likely originating from the soils’ parent material (lithogenic origin). As and Hg are likely derived from the traditional extraction process of gold ore, amalgamation for gold extraction [27,59].

The results obtained from PCA (Table 3) for PTEs in sediments showed that F1 explained 42.7% of the total variance with high positive loadings, Cd (0.850), Cu (0.820), Ni (0.753), Pb (0.722), Zn (0.685), and Cr (0.593), meaning a common source is possibly mining activities; F2 with a high value for Cr (0.733) described 26.6% of the total variance. Both F1 and F2 contained Cr, suggesting that Cr could originate from multiple sources. This speculation is further supported by the HCA and Pearson’s correlation coefficient. The HCA results divided the PTEs into two groups in the sediments: As, Hg, Pb, Zn, and Cd in group 1; and Cu, Cr, and Ni in group 2, indicating that the same group may originate from the common source(s) (Figure 5). In addition, Cu-Cr-Ni and Zn-Cd were significantly correlated with each other (r < 0.6, *p* < 0.01) based on Pearson’s correlation analysis. Thus, it can be observed that the correlation of PTEs in sediments is weaker compared to soils. At the same time, the overall content of PTEs is low, and fewer sampling sites are affected by mining activity disturbance.

### 3.4. Pb Isotope Ratios and Source Apportionment

**Figure 6:**
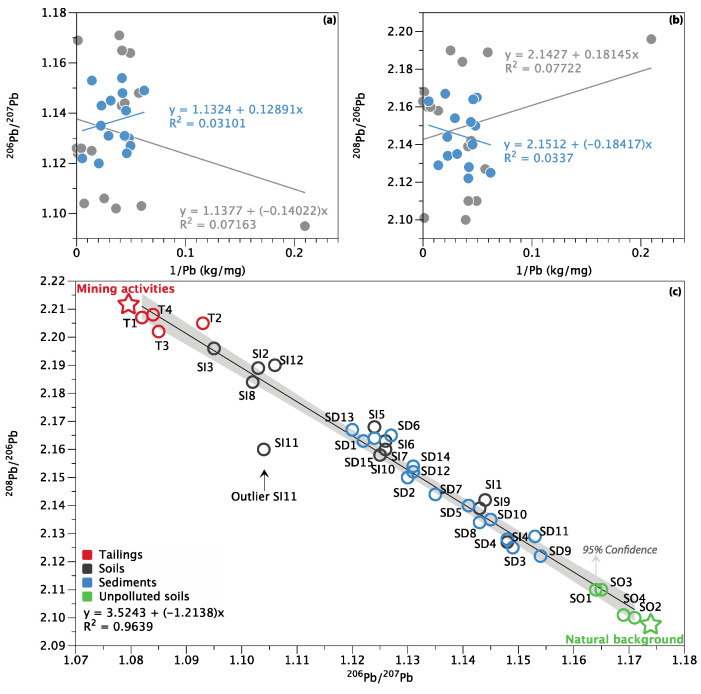
The plot of ^206^Pb/^207^Pb (**a**) and ^208^Pb/^206^Pb (**b**) versus the inverse of Pb concentration of the soils and sediments, as well as the plot of ^206^Pb/^207^Pb versus ^208^Pb/^206^Pb ratios of the sediments, soils, and tailings in this study (**c**).

As shown in Figure 6, the uncorrelated relationship between Pb concentration (1/Pb, kg/mg) and Pb isotopic ratio (^206^Pb/^207^Pb and ^208^Pb/^206^Pb) in both soils (R^2^ = 0.07) and sediments (R^2^ = 0.03) indicated a mixing of different Pb sources in soils and sediments (Xu et al., 2019b). The plot of ^206^Pb/^207^Pb vs. ^208^Pb/^206^Pb ratios of the soils, sediments, and tailings in this study is displayed in Figure 6c. The Pb isotopic composition of soils outside the mining area (SO1–SO4) received a significant input of adventitious Pb with high ^206^Pb/^207^Pb (1.167 ± 0.0029) and low ^208^Pb/^206^Pb (2.105 ± 0.0048), which is in line with China soils from the northeast geochemical region ^206^Pb/^207^Pb of 1.153–1.175, and ^208^Pb/^206^Pb ratios of 2.11 ± 0.005 [60]. Pb found in unpolluted sediments was usually derived from various natural sources, including weathering of catchment soils and bedrock or transported more directly within mineral matter eroded from catchment [61]. As listed in Table 4, there was a wider range of ^206^ Pb/^207^Pb and ^208^ Pb/^206^Pb ratios in soils (1.095–1.148 and 2.127–2.196) compared to sediments (1.120–1.154 and 2.122–2.167), which is consistent with data obtained in the corresponding Pb concentration. This reflected that soils within the mining area may be more severely disturbed by mining activities than sediments are. The dominating sources of Pb pollution in the Chinese mining area may originate from mining and industrial emissions such as metal processing and manufacturing, as well as coal combustion (transportation of aerosol deposition) and vehicle exhausts [30,47,53]. The contribution of leaded gasoline was not considered in this study, because, at the end of the last century, leaded gasoline was completely banned in China, and leaded gasoline has a quite low Pb concentration [16].

**Figure 7:**
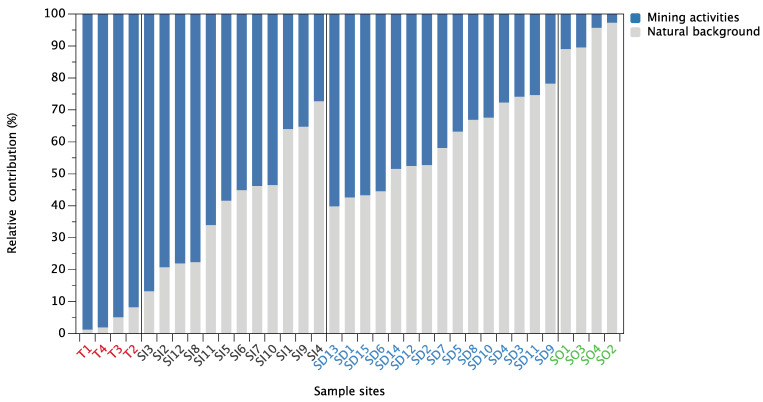
Average relative contribution (%) of Pb from different sources in soils and sediments.

Given a strong linear trend between the Pb isotope ratios (^208^Pb/^206^Pb vs. ^206^Pb/^207^Pb) of tailings, unpolluted soils, soils, and sediments (R^2^ = 0.96), only the source contributions of Pb from mining activities (as source A) and natural background (as source B) were considered in this study, and the average relative Pb contributions were calculated for each site according to Equations (6)–(8). As shown in Figure 7, the results showed that mining activity contributes most of the mining activity-related Pb to soils, with an average relative contribution of 58.9%, ranging from 27.2% (SI4) to 86.7% (SI3). For the sediments, the natural background appeared to be the main source of Pb (58.8%), while contributions from mining activity ranged from 21.7% (SD9) to 60.2% (SD13). It confirmed that mining activity was the major source of Pb pollution in soils. This phenomenon is mainly due to the considerable contribution of long-term frequent mining activities, ore mining and smelting, and abundant small-scale mines distributed in the upper area of the Miyun Reservoir; therefore, most soil Pb likely represents locally emitted Pb [27,30,33,62]. In contrast, natural background sources are an important source of Pb in sediments. Nevertheless, some of the sample sites (e.g., SD13, SD1, SD15, and SD6) are still strongly disturbed by mining activities, with the contribution of mining activities greater than 50%. Several pieces of research have also suggested that mining activities were the dominant anthropogenic Pb source in reservoir sediments [63,64]. It is noteworthy that mining activity-related Pb sources account for 66% of the significant outliers (SI11) in Figure 6. It is speculated that the reason for the deviation may be the influence of other external sources at this sample site, which significantly changed its Pb isotopic composition. This is also supported by the HCA results that, although both sample sites SI6 and SI11 are heavily contaminated (Table 2), the dominant PTEs are significantly different (Figure 5).

## 4. Conclusions

Soils and sediments around the gold mine site have been affected to varying degrees by mining activities, and the soils have been more strongly disturbed. The average concentrations of PTEs in the soil markedly exceeded the local background values, and the Pb content in some sample sites was even several hundred times higher than the background values. The results of the multivariate statistical analysis suggested that the accumulation of Cu, Zn, Pb, and Cd may be mainly from mining activities, while Cr and Ni are from natural background sources in soils. Soils have much wider Pb isotopic ratio (^208^Pb/^206^Pb and ^206^Pb/^207^Pb) ranges than sediments do in the study area. The results of Pb isotopic fingerprinting with a binary mixing model indicated that the average relative contribution of mining activities to Pb accumulation accounts for 58.9% in soils and 41.2% in sediments. The mining activities were suggested to be the dominant contribution of Pb pollution in soils. The findings provide quantitative guidance for the environmental management of PTEs and control of the mining activities around the Miyun Reservoir.

## Figures and Tables

**Table 1:** Concentration of PTEs in soils, unpolluted soils, and sediments.

Element	Cu	Pb	Zn	Cr	Ni	Cd	As	Hg
	mg/kg							
Soils (*n* = 36)								
Max	1580	27400	792	195	62.3	4.10	59.1	1.140
Min	13.0	4.77	18.7	44.3	18.2	0.0970	2.07	0.004
Mean	199	2470	181	117	40.4	0.791	8.52	0.168
Meadium	71.4	55.7	93.2	105	38.4	0.315	3.92	0.026
SD	438	785	211	52.7	13.8	1.12	16.0	0.329
CV%	220	31.8	116	45.2	34.1	141	188	197
Unpolluted Soils (*n* = 12)								
Max	29.0	25.5	71.4	80.1	33.9	0.150	7.45	0.000019
Min	19.4	20.3	49.7	43.8	19.5	0.110	2.49	0.000010
Mean	23.6	23.1	64.2	55.7	24.9	0.128	4.71	0.000014
Meadium	23.0	23.3	67.9	49.5	23.2	0.125	4.46	0.000014
SD	4.79	2.21	9.83	17.0	6.87	0.0171	2.10	0.000004
CV%	20.3	9.57	15.3	30.5	27.6	13.4	44.5	28.0
Sediments (*n* = 45)								
Max	92.1	192	325	129	45.5	0.910	15.0	0.000190
Min	16.7	16.1	46.9	44.7	18.4	0.100	2.46	0.000010
Mean	36.7	42.5	113	68.8	28.9	0.336	5.10	0.000034
Meadium	29.8	24.0	100	64.2	27.1	0.200	4.29	0.000017
SD	19.3	43.9	74.3	22.6	8.51	0.262	3.15	0.000044
CV%	52.7	103	66	32.8	29.4	77.8	61.7	129
BBV ^a^	22.5	23.7	71.4	80.1	33.9	0.20	7.50	0.0700
UCC ^b^	28.0	17.0	67.0	92.0	47.0	0.0900	4.80	0.0500

^a^ Beijing Background Value; ^b^ Upper Continental Crust.

**Table 2:** Pollution assessment of PTEs in soils and sediments.

Type	Site	Contamination Factor (*CF*)								Pollution Load Index (*PLI*)		Potential Ecological Risk Index (RI)	
		Cu	Pb	Zn	Cr	Ni	Cd	As	Hg	Values	Pollution Levels	Values	Pollution Levels
Soils	SI1	5.20	35.4	3.50	2.23	1.53	5.30	0.36	0.34	2.48	Moderate	381	Considerable
	SI2	1.72	0.71	1.40	2.43	1.84	1.15	0.41	0.11	0.90	Unpolluted	66	Low
	SI3	0.58	0.20	0.26	2.35	1.04	0.60	0.58	0.06	0.44	Unpolluted	38	Low
	SI4	4.53	0.74	0.86	1.31	1.50	0.65	0.79	0.19	0.93	Unpolluted	65	Low
	SI5	4.49	37.0	3.31	1.30	1.23	4.80	0.28	0.36	2.12	Moderate	366	Considerable
	SI6	70.3	1150	11.1	1.92	1.06	20.5	0.55	3.97	9.75	Very high	6767	High
	SI7	3.61	9.79	3.92	1.30	1.15	6.05	0.40	0.53	2.01	Moderate	265	Moderate
	SI8	2.04	1.16	0.90	0.80	0.90	0.49	0.29	0.14	0.65	Unpolluted	40	Low
	SI9	0.89	1.02	0.78	0.59	0.65	0.85	0.83	0.39	0.72	Unpolluted	49	Low
	SI10	2.27	3.03	1.21	0.55	0.54	2.00	0.50	5.46	1.38	Moderate to unpolluted	98	Low
	SI11	7.64	6.03	2.07	1.57	1.76	4.05	7.88	16.3	4.39	High	286	Considerable
	SI12	2.73	1.67	1.08	1.13	1.11	1.05	0.79	0.93	1.21	Moderate to unpolluted	70	Low
Unpolluted soils	SO1	1.29	0.86	0.94	1.00	1.00	0.75	0.67	0.27	0.78	Unpolluted	48	Low
	SO2	0.88	1.08	1.00	0.55	0.58	0.65	0.33	0.14	0.56	Unpolluted	38	Low
	SO3	1.16	1.01	0.96	0.68	0.79	0.60	0.99	0.21	0.73	Unpolluted	45	Low
	SO4	0.86	0.95	0.70	0.56	0.58	0.55	0.52	0.17	0.56	Unpolluted	36	Low
Sediments	SD1	4.09	8.10	1.99	1.05	0.89	2.50	0.33	0.24	1.37	Moderate to unpolluted	148	Low
	SD2	1.77	0.88	1.61	0.82	0.75	0.75	0.539	0.23	0.78	Unpolluted	48	Low
	SD3	2.30	0.68	0.66	1.61	1.27	0.75	0.49	0.21	0.80	Unpolluted	53	Low
	SD4	2.59	1.00	0.89	1.31	1.34	1.00	0.59	0.19	0.90	Unpolluted	64	Low
	SD5	1.22	0.93	0.83	0.62	0.66	0.90	0.57	0.33	0.71	Unpolluted	49	Low
	SD6	0.74	0.85	0.83	0.75	0.54	0.50	0.55	0.20	0.57	Unpolluted	34	Low
	SD7	1.23	1.89	1.07	0.84	0.90	2.30	0.34	0.14	0.81	Unpolluted	95	Low
	SD8	1.32	1.85	1.62	0.69	0.73	1.55	0.53	2.64	1.20	Moderate to unpolluted	75	Low
	SD9	1.03	1.01	1.24	0.58	0.61	0.85	0.60	0.51	0.77	Unpolluted	47	Low
	SD10	1.70	1.35	1.57	0.75	0.90	1.95	0.78	0.89	1.16	Moderate to unpolluted	89	Low
	SD11	1.90	3.00	4.55	0.94	1.20	4.55	2.00	0.70	1.92	Moderate to unpolluted	194	Moderate
	SD12	1.04	0.96	0.74	0.56	0.63	0.50	0.65	0.14	0.57	Unpolluted	36	Low
	SD13	1.54	2.07	3.19	0.80	1.00	4.45	1.19	0.46	1.45	Moderate to unpolluted	173	Moderate
	SD14	1.18	1.44	1.40	0.86	0.87	1.75	0.67	0.29	0.94	Unpolluted	80	Low
	SD15	0.81	0.92	1.44	0.73	0.62	0.90	0.38	0.19	0.65	Unpolluted	45	Low

**Table 3:** Results of principal component analysis.

Element	Soils		Sediments	
	F1	F2	F1	F2
Cu	**0.938**	0.046	**0.820**	0.417
Pb	**0.943**	−0.169	**0.722**	−0.228
Zn	**0.943**	−0.062	**0.685**	−0.600
Cr	0.234	**0.904**	**0.592**	**0.733**
Ni	0.225	**0.924**	**0.753**	0.487
Cd	**0.952**	−0.054	**0.850**	−0.365
As	0.222	0.223	0.102	−0.468
Hg	**0.706**	−0.343	0.325	−0.643
Eigenvalue	4.22	1.88	3.42	2.13
Variability (%)	52.7	23.4	42.7	26.6
Cumulative (%)	52.7	76.2	42.7	69.3

Greater than 0.5 are shown in bold.

**Table 4:** Pb isotopic component in soils and sediments.

Type	Sample Site	^208^Pb/^204^Pb	^207^Pb/^204^Pb	^206^Pb/^204^Pb	^206^Pb/^207^Pb	^208^Pb/^206^Pb
Soils	SI1	37.92	15.48	17.70	1.144	2.142
	SI2	36.72	15.21	16.77	1.103	2.189
	SI3	36.77	15.30	16.75	1.095	2.196
	SI4	37.78	15.47	17.76	1.148	2.127
	SI5	38.04	15.61	17.55	1.124	2.168
	SI6	37.91	15.56	17.52	1.126	2.163
	SI7	37.61	15.47	17.41	1.126	2.160
	SI8	36.91	15.34	16.90	1.102	2.184
	SI9	37.81	15.47	17.68	1.143	2.139
	SI10	37.51	15.45	17.38	1.125	2.158
	SI11	36.27	15.21	16.79	1.104	2.160
	SI12	37.15	15.33	16.96	1.106	2.190
	mean	37.37 ± 0.558	15.41 ± 0.124	17.26 ± 0.382	1.121 ± 0.0175	2.165 ± 0.0210
	median	37.56	15.46	17.40	1.125	2.162
Unpolluted soils	SO1	38.21	15.55	18.11	1.164	2.110
	SO2	38.21	15.54	18.19	1.171	2.100
	SO3	38.15	15.52	18.08	1.165	2.110
	SO4	38.15	15.54	18.16	1.169	2.101
	mean	38.18 ± 0.030	15.54 ± 0.011	18.14 ± 0.043	1.167 ± 0.0029	2.105 ± 0.0048
	median	38.18	15.54	18.14	1.167	2.106
Sediments	SD1	37.48	15.45	17.33	1.122	2.163
	SD2	37.47	15.42	17.42	1.130	2.150
	SD3	37.82	15.48	17.79	1.149	2.125
	SD4	37.79	15.47	17.76	1.148	2.128
	SD5	37.80	15.48	17.66	1.141	2.140
	SD6	37.66	15.43	17.39	1.127	2.165
	SD7	37.67	15.48	17.57	1.135	2.144
	SD8	37.76	15.48	17.69	1.143	2.134
	SD9	37.90	15.47	17.85	1.154	2.122
	SD10	37.84	15.48	17.73	1.145	2.135
	SD11	37.99	15.49	17.85	1.153	2.129
	SD12	37.58	15.43	17.46	1.131	2.152
	SD13	37.42	15.41	17.27	1.120	2.167
	SD14	37.57	15.42	17.44	1.131	2.154
	SD15	37.52	15.430	17.34	1.124	2.164
	mean	37.68 ± 0.168	15.45 ± 0.027	17.57 ± 0.196	1.137 ± 0.0111	2.145 ± 0.0152
	median	37.67	15.47	17.57	1.135	2.144
Tailings	T1	36.63	15.30	16.60	1.085	2.207
	T2	36.69	15.23	16.64	1.093	2.205
	T3	36.53	15.30	16.60	1.085	2.202
	T4	36.63	15.24	16.60	1.085	2.208
	mean	36.62 ± 0.057	15.27 ± 0.033	16.61 ± 0.017	1.087 ± 0.0035	2.205 ± 0.0023
	median	36.63	15.27	16.60	1.085	2.206
